# Femoral neck anteversion, acetabular anteversion and combined anteversion in the normal Indian adult population: A computed tomographic study

**DOI:** 10.4103/0019-5413.65156

**Published:** 2010

**Authors:** Aditya V Maheshwari, Michael P Zlowodzki, Gautam Siram, Anil K Jain

**Affiliations:** 1 Department of Orthopedics, University College of Medical Sciences, University of Delhi – 110 095, India; 2 Department of Orthopedics, Washington Hospital Center, 110 Irving ST NW, Washington DC, USA - 200 10; 3 Department of Orthopedics, University of Minnesota, R200, 2450 Riverside Ave S, Minneapolis, MN – 554 54; 4 Department of Orthopedics, Howard University Hospital, 2041 Georgia Avenue, Washington, DC –20060

**Keywords:** Acetabular anteversion, combined anteversion, computed tomography, developmental dysplasia of the hip, femoral neck anteversion, hip anthropometry, hip impingement, Indian hips, proximal femoral morphology, total hip replacement

## Abstract

**Background:**

Abnormal femoral neck anteversion (FNA) and/or acetabulum anteversion (AA) have long been implicated in the etiogenesis of hip osteoarthritis (OA), developmental dysplasia of the hip (DDH), and impingement, instability and wear in total hip arthroplasty (THA). Since studies on the Indian population are sparse on this topic, the purpose of this study was to report the normal values of FNA, AA and the combined anteversion (CA= FNA+ AA) in Indian adults.

**Materials and Methods:**

FNA, AA and CA were prospectively measured in 172 normal hips in 86 Indian adults using standardized computed tomographic (CT) methods and this data was compared with the established Western values.

**Results:**

The median values and interquartile ranges were 8° (6.5-10.0°) for FNA, 19° (16.0-22.0°) for AA and 27° (23.5-30.0°) for CA. AA and CA values were significantly (*P*<0.05) lower in males, and there was also a trend towards lower FNA in males. Although a negative correlation was observed between the FNA and AA, this was not strong and may not be clinically relevant.

**Conclusion:**

When compared with the Western data, the FNA values were 3-12° lower and the CA values were 3-5° lower in Indian adults. The AA values were comparable, but were skewed towards the higher side. Further studies are needed to assess the clinical relevance of our basic science data in pathogenesis of OA, and to validate it in relation to hip surgeries like corrective osteotomies and THA.

## INTRODUCTION

The mechanics of the hip joint are dependent on the relationship between the femoral head and the acetabulum.^1^–^9^ Abnormal femoral neck anteversion (FNA) and/or acetabulum anteversion (AA) have long been implicated in the etiogenesis of osteoarthritis, developmental dysplasia of the hip (DDH), and impingement, instability and wear in total hip arthroplasty (THA).^1^–^9^ Combined anteversion (CA) or the ‘instability index’ of the hip has been defined as the sum of the FNA and the AA (CA= FNA+AA).^2^,^10^ Although the concept of CA was described in the early 20^th^ century for DDH,^7^ it has gained popularity in recent years in THA literature.^10^–^20^

A review of the global literature reveals a wide range of normal FNA and AA with racial and geographic variation.^1^,^3^,^21^–^38^ This variation is expected to exist because of different social needs of the different races. Numerous studies have focused on FNA in the normal population; however, little attention has been given to the normal AA and the CA. Moreover, studies on the Indian population are sparse on this topic.^32^–^34^ Since Indians are more prone to indulge in floor level activities like squatting and sitting cross-legged, the hip is flexed, externally rotated and abducted to the extremes of motion. We were interested whether this resulted in morphologically different hip anteversion in Indians as compared to the Western population. In previous studies, we had reported the preliminary data on normal FNA in Indian adults and had compared and contrasted our data with other Western studies.^23^–^26^ The purpose of this study was to update our data on FNA, define a normal range of values for FNA, AA and CA for the Indian adults, and to investigate the relationship of FNA and AA.

## MATERIALS AND METHODS

After Institutional review board approval, all consecutive adults who had a computed tomographic (CT) scan of the pelvis/thigh for pathology unrelated to the hip during 2002-05, were considered for this prospective study. The exclusion criteria were (1) patients with bony pathology of the pelvis and femur, (2) patients with hip pathology as evident clinically with gait abnormality and/or pain/restriction of hip motions, (3) prior surgical intervention, (4) childhood hip, knee or spine disease, (5) patients with current or previous metabolic bone disease, and (6) uncooperative patients. All patients had current serum calcium, phosphate and alkaline phosphatase levels.

**Figure 1:**
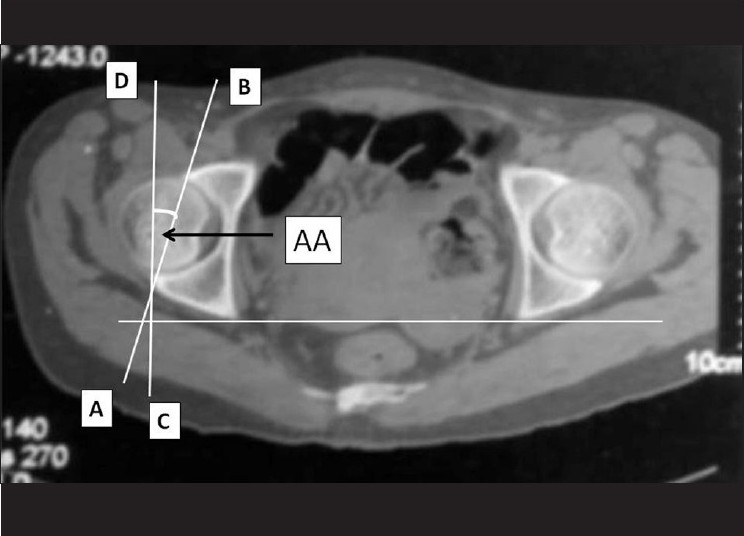
On an axial CT scan through the center of the acetabulum, a line was drawn between the anterior and posterior edges of the acetabulum (AB) and the angle between this line and a plane sagittal to the pelvis (CD) was determined to be acetabular anteversion

All patients were evaluated using a Quad Slice Siemens Somatome Plus 4 Volume Zoom CT scanner (Siemens, Germany). The method of measuring the FNA has been described earlier.^23^,^26^,^39^,^40^ We used the central axis of the neck and the posterior condylar axis as our references. We did not use the center of the head for the neck axis on CT^39^ as the majority of the femoral heads are not in the center of the femoral neck.^21^–^26^,^37^ The posterior condylar axis was used as it has been shown to be the most reproducible, not only on the same image, but also on separate images.^39^ This method also has the advantage of theoretically correlating with the dry bone method and clinicoradiological methods of measurement, whereby the knee is flexed to 90°, the tibia is vertical, and the condylar plane is assumed to be horizontal.^22^–^26^,^41^ The estimation of the AA was done by the method described by Reikeras *et al*.^3^ On a scan through the center of the acetabulum, a line was drawn between the anterior and posterior edges of the acetabulum and the angle between this line and a plane sagittal to the pelvis was determined to be AA [Figure 1]. The CA was then calculated as (AA+FNA).

Measurements were done twice by a single observer (AVM) at a minimum interval of one week. Intraobserver reliability was calculated by comparing the two independent measurements by the same person (AVM); interobserver reliability was the comparison of the measurements between the two observers (AVM and MPZ). Only the mean of readings by one observer (AVM) was used for all other analysis.

### Statistical analysis

Data was analyzed with the SPSS/PC + statistical package (SPSS Version 16.0, Chicago, IL). Distribution of variables for each group was tested for normality using the Kolmogorov-Smirnov test. Since these were normally distributed variables, the differences between the sides (right and left) and the gender (male and female) were estimated using a paired t-test and an unpaired t-test respectively. A Pearson correlation coefficient was calculated between FNA and AA. We also determined the inter and intra-observer differences in the measurements with a paired t-test and the associations with an intra-class correlation coefficient (ICC). We also included the mean ± 5° data in our analysis for each variable as accuracy within 10° is considered adequate clinically^31^ and in recent navigated THA studies, the precision of surgeon’s estimate of component anteversion when compared to a CT scan has been shown to be within 11°.^10^–^12^

## RESULTS

A total of 90 patients met the eligibility criteria. Four patients later withdrew from the study and were excluded from analysis. Thus we studied 172 hips in 86 patients. There were 40 male and 46 females with a median age of 33 years (range, 18-70). The results of FNA in 36 of these patients have been described in our earlier report.^23^,^26^

**Figure 2:**
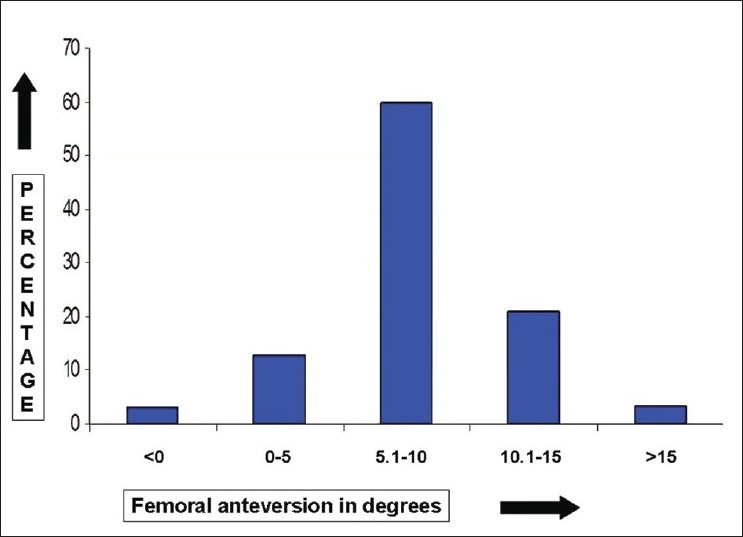
A bar diagram showing the distribution of femoral neck anteversion in normal Indian adults

**Figure 3:**
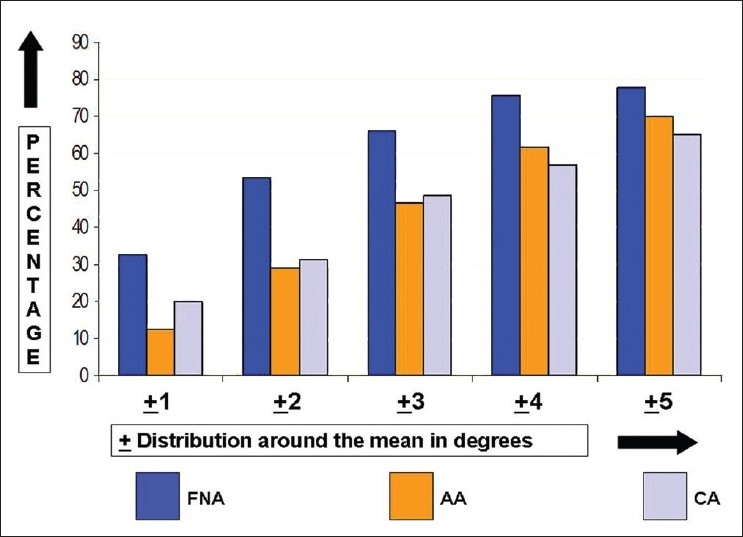
A bar diagram showing the distribution of femoral neck anteversion, acetabular anteversion and combined anteversion (acetabular anteversion + femoral neck anteversion) as ± 5° of their respective means

**Figure 4:**
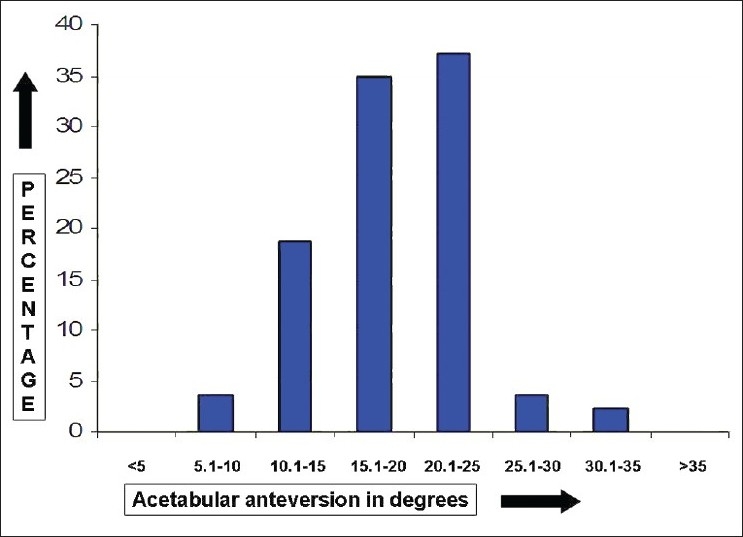
A bar diagram showing the distribution of acetabular anteversion in normal Indian adults

**Figure 5:**
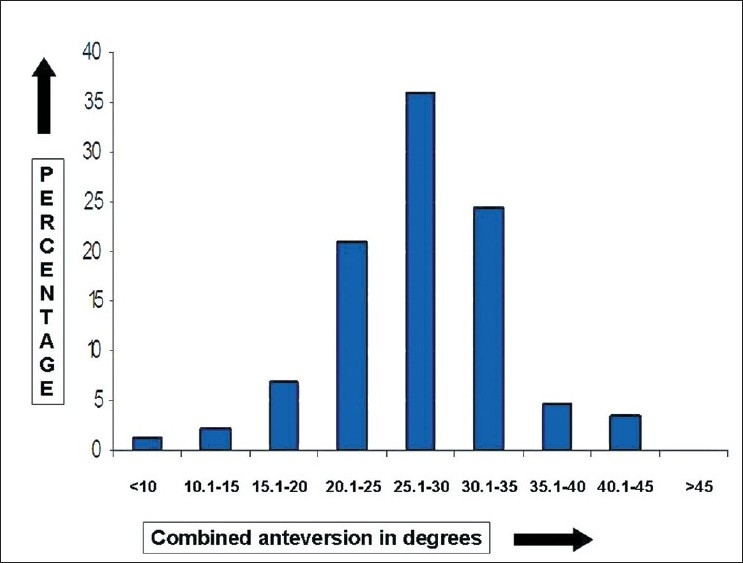
A bar diagram showing the distribution of combined anteversion (acetabular anteversion + femoral neck anteversion) in normal Indian adults

The mean FNA was 8.0° (median 8.0°, standard deviation (SD) 4.7°, range 12.0-22.0°, interquartile range 6.5°-10.0°); 59.8% of the study cohort had FNA between 5°-10° and 77.9% had FNA within ±5° of the mean [Figures 2 and 3]. The mean AA was 19.1° (median 8.0°, SD 5.0°, range 8.0°-35.0°, interquartile range 16.0°-22.0°); 72.1 % of the cohort had AA between 15°-25° and 69.8% had AA within ±5° of the mean [Figures 3 and 4]. The mean CA was 27.1° (median 27°, SD 6.3°, range 9.5°–43.0°, interquartile range 23.5°–30.0°); 81.3 % of the cohort had CA between 20°-35° and 65.1% had CA within ±5° of the mean [Figures 3 and 5].

The difference between the genders and sides is shown in Table 1. An inverse but not clinically strong correlation (-0.2) was seen between FNA and AA. Although 80 (93%) of the patients were right-hand dominant, no correlation was found between the handedness (right vs. left) and the FNA, AA or the CA. No correlation was found between the age of the patient and the FNA, AA or the CA. We observed no differences in the mean inter (*P* = 0.8) or intra-observer (*P*=0.9) measurements: the mean inter-observer difference was 0.1° (SD 1.2°, range -3° to 3°) and the mean intra-observer difference was 0.1° (SD 0.8°, range -2° to 2°). The ICC for both inter and intra-observer difference was 0.9 (*P*=0.001).

**Table 1:** Gender and side difference between femoral neck anteversion (FNA), acetabular anteversion (AA) and the combined anteversion (CA)

	*n* (hips)	Mean FNA	*P* value	Mean AA	*P* value	Mean CA	*P* value
Males	80	7.3°	0.17	17.3°	0.001*	24.6°	0.001*
Females	92	8.7°		20.8°		29.5°	
Right	86	7.4°	0.03*	19.2°	0.45	26.6°	0.19
Left	86	8.7°		18.9°		27.6°	
Total	172	8.0°		19.1°		27.1°	

* Statistically significant difference

## DISCUSSION

The morphology of the hip joint has always interested the orthopedic community. Although numerous studies have focused on FNA in the normal population, relatively little attention has been given to the normal AA and the CA. This may be due to the relatively complex anatomy of the pelvis and lack of unanimity in defining a reference plane.^2^,^35^ In addition, a wide range of racial and geographic variations has been documented due to the different lifestyles and social needs of different races.^1^,^3^,^21^–^38^,^42^ Most of the available data on this topic is from studies of Western populations, whereas studies on the Indian population are sparse.^32^–^34^ Although three recent Indian studies discuss the normal FNA,^32^–^34^ to the best of our knowledge, no previous study has correlated the FNA, AA and the CA in normal Indian adults along with comparison with the Western literature.

The estimation of anteversion on dry bones may be considered most accurate, but inclusion of some pathologic bones may influence the statistical analysis, thus questioning its relevance for clinical practice.^21^,^25^ Of the various radiologic methods, the CT method is considered to be ±1° accurate as tested on the specimens and thus was used in this study.^39^ The mean FNA in our study was 8.0° with a wide range (-12° to 22°) of distribution. This is similar to our previous studies using a CT scan on 36 patients (mean 7.4°) and 300 dry femora (mean 8.1°).^23^–^26^ When compared to previous Western data using a similar CT scan methodology, the FNA in our study population was 3-12° lower.^1^,^3^,^29^–^31^,^38^ Our data is skewed towards a lower value and more than 96% of the values were less than 15° (15° is an acceptable mean in most Western studies).^1^,^3^,^29^–^31^,^38^,^42^ Interestingly, our mean values are also less than other Indian studies.^32^–^34^ A recent CT study on 92 North-East Indians estimated the normal FNA as 20.4° (8-45°, SD 5.4°), AA as 18.2° (8-40°, SD 5.5°) and thus the CA as 38.6°.^32^ Siwach *et al*.,^34^ studied 150 dry femora and found the mean FNA as 13.7° (0-36°, SD 7.9°). Nagar *et al*.,^33^ studied 182 dry femora and found the mean FNA as 11.3° ± 0.4° and 21.°2 ± 0.4° on the left and right sides respectively in males, and 11.0° ± 0.3° and 20.9° ± 0.4° on the left and right sides respectively in females. Although it is difficult to explain these differences, the use of a different reference axis (head center in Saikia *et al*.,^32^ and Nagar *et al*.,^33^ and transcondylar axis in Siwach *et al*.^34^ ) can account for a difference of up to 6°.^30^,^38^ In our opinion, a mean FNA of 20.4° as in Saikia *et al*.’s study^32^ and an incredible difference of 10° between the left and the right side in Nagar *et al*.’s^33^ study needs further scrutiny as clinical experience has never shown the normal FNA in this range. These figures appear abnormal and would likely lead to a gait abnormality.

The mean AA in our study was 19.1°, again with a wide range (8.0°-35.0°). Less unanimity is present in the literature about the normal AA values, which range from 15-42°.^1^–^3^,^27^,^42^ This has been attributed to a lack of consistent reference planes as defined by Murray.^35^ Using his definition recent western studies have shown the mean AA to be 15-20°.^1^,^3^,^27^,^42^ Although, the Western mean appears quite comparable to our study, our data is skewed towards the higher side and 78% of our patients had an AA of more than 15°.

The mean CA in our study was 27.1° (range 9.5°–43.0°). This is 3-5° lower compared to other Western studies.^3^,^27^ Thus our data is skewed towards the lower side and 81.3% of the hips had CA between 20-35°. This is not surprising considering the fact that FNA is lower in the Indian population, while AA is comparable or slightly on the higher side as compared to the Western data. However, this also suggests that lower FNA value (rather than AA) is the major determinant in explaining this difference in the CA, a finding which has been previously described by Reikeras *et al*. in osteoarthritis (OA) of the hip.^3^ Although a negative correlation was observed between the FNA and AA, this was not strong and is in concordance with a previous study.^3^ Thus abnormal FNA or AA may not be compensated by each other. The finding also supports the evaluation of the hip using the CA values, rather than just the individual FNA and AA values as was usually done in the past.

Gender and side differences in FNA have been noted in numerous previous studies.^3^,^21^,^23^,^25^–^27^,^33^ Although males tend to have less FNA as compared to females, this did not reach statistical significance in this study. Moreover, we also noted a significant difference between the left and the right side, the latter being lower. We did not find any correlation of handedness of the person with FNA as this seemed the most logical explanation of side difference. On the other hand, females had significantly higher values for AA and CA in our study and this is in agreement with the literature.^3^,^27^

So what may be the possible clinical implications of these differences between the Indian and the Western data? Previous studies have demonstrated the role of increased FNA and CA in the pathogenesis of primary OA of the hip,^3^–^6^,^8^ presumably due to uncovering and unequal distribution of forces at the hip. Although we do not know the exact incidence of primary hip OA in the Indian population, experience tells us that it is much lower when compared to the Western population. Similarly, females have a higher incidence of hip OA and females have a less favorable relationship at the hip as evident by increased FNA, AA and thus the CA.^3^ On the other hand, Tonnis and Heinecke^1^ postulated that a CA of less than 20° in patients with childhood hip disorders was a major cause of hip pain, decreased range of motion and OA. Almost 90% of our study cohort had a CA of more than 20° and 85.9% of the values were between 20-40°. Are these mid-values of FNA and CA protective (in terms of a ‘safe zone’) in terms of primary hip OA in Indians? This is difficult to prove from our study but the relationship of the hip anteversion angles and the prevalence of primary hip arthritis needs further investigation in the Indian population.

The other implication of our data may be in THA as component positioning is important to minimize impingement, instability and subsequent wear.^12^,^15^ The concept of using CA, rather than ‘target values’,^43^–^46^ to determine the cup position when mating it with the uncemented stem is becoming more prevalent in recent times.^10^–^20^ This is because the stem anteversion cannot be controlled as opposed to a cemented hip and the fact that the native FNA may have a wide range of distribution.^10^–^15^ Komeno *et al*.,^19^ concluded that the dislocation rate is not affected by the positioning of either the cup or the stem alone but is influenced by the CA. Excessively increased CA can lead to anterior dislocation and excessively decreased CA can lead to posterior dislocation. McKibbin^2^ defined the normal instability index (FNA + AA) for anatomic hips to be 30-40°, with a range of 20-35° for men and 30-45° for women. Ranawat and Maynard^16^ recommended a CA of approximately 45° in females and 20-30° in males and Ranawat^11^,^13^,^15^ has described a test to evaluate the CA peroperatively. Using computer navigation, Dorr *et al*.,^10^–^15^ recommended a CA of 25-49°. These recommendations are interesting as about 90% of our study population had a CA of 20-45°. However, we need to keep in mind that there may be some difference between the native hip and the THA due to different natural and mechanical constraints as well as a different head-neck ratio. That’s why we need further studies on THA as well to recommend our target CA for the Indian population.

A limitation of this study is its relatively small sample and much larger studies, preferably multicenter ones, would be needed to expand the Indian database. Still this is one of the largest series of its kind. We also did not account for the ethnic variation, which is common in metropolitan areas. This study is just a snapshot at a point in time. Although these patients appeared normal at the time of this study, we still do not know how they will fare in terms of their hips in the future, when some of the extreme values may no longer appear normal. It is also important to consider the methods of evaluation while comparing our data with other studies as different methods may give differing results.^30^,^38^ This was a basic science study and further studies are needed to assess the clinical relevance of this data in the pathogenesis of OA, and to validate it in relation to hip surgeries like corrective osteotomies and THA.
